# Targeting TLR2 for Vaccine Development

**DOI:** 10.1155/2014/619410

**Published:** 2014-06-26

**Authors:** Afonso P. Basto, Alexandre Leitão

**Affiliations:** ^1^Centro de Investigação Interdisciplinar em Sanidade Animal (CIISA), Faculdade de Medicina Veterinária, Universidade de Lisboa, Avenida da Universidade Técnica, 1300-477 Lisboa, Portugal; ^2^Instituto de Investigação Científica Tropical, CVZ, FMV, Avenida da Universidade Técnica, 1300-477 Lisboa, Portugal

## Abstract

Novel and more effective immunization strategies against many animal diseases may profit from the current knowledge on the modulation of specific immunity through stimulation of innate immune receptors. Toll-like receptor (TLR)2-targeting formulations, such as synthetic lipopeptides and antigens expressed in fusion with lipoproteins, have been shown to have built-in adjuvant properties and to be effective at inducing cellular and humoral immune mechanisms in different animal species. However, contradictory data has arisen concerning the profile of the immune response elicited. The benefits of targeting TLR2 for vaccine development are thus still debatable and more studies are needed to rationally explore its characteristics. Here, we resume the main features of TLR2 and TLR2-induced immune responses, focusing on what has been reported for veterinary animals.

## 1. Introduction

The innate immune system senses microorganisms through germ-line encoded receptors, the pattern recognition receptors (PRRs), which include the membrane associated toll-like receptors (TLRs) [1]. Based on the knowledge that stimulation of PRRs by pathogen-associated molecular patterns (PAMPs) has a determinant role in shaping the profile of the subsequent adaptive immune response [2, 3], the conjugation of antigens with PRR ligands has been extensively explored in the last decades for the development of improved vaccines [4–7]. For that purpose, much attention has been paid to PRR ligands inducing strong polarized Th1 and cytotoxic T lymphocyte (CTL) responses, for example, ligands for TLR3, TLR7/8, or TLR9, since these are immune mechanisms poorly induced by vaccination with nonlive, inactivated or subunitary, vaccines. Activation through TLR2 is not recognized as a strong polarizing stimulus, resulting in Th responses with variable characteristics. However, TLR2 offers unique properties to be explored in vaccine development. The possibility to covalently attach TLR2 ligands to antigens, the enhancement of direct- and cross-presentation of antigens coupled to TLR2-targeting lipid moieties, the capacity to induce balanced Th responses and even regulatory mechanisms, and the mucosal imprinting properties of TLR2 stimulation are characteristics that have potential to help solving actual vaccine challenges. Here, we will review the present knowledge on the modulation of the immune response by immunogenic formulations targeting TLR2 and discuss its potential for the development of immunization strategies in the veterinary field.

## 2. TLR2

### 2.1. The Receptor

TLRs are transmembrane type I glycoproteins with a structure composed by three domains. The N-terminal extracellular domain, which is involved in the recognition of their ligands, consists of leucine-rich repeats (LRR) with the conserved motif “LxxLxLxxN” with around 20 to 30 amino acids. This domain is followed by a transmembrane region then extended intracellularly by a cytoplasmic toll/IL-1 receptor (TIR) domain, needed for signal transduction [1, 8, 9].

Phylogenetically, TLR2 belongs to a TLR family that includes TLR1, TLR6, TLR10, TLR14, and possibly the avian TLR15 [10]. TLR2 is located at the surface of the cell and, upon binding of its ligands, dimerises with TLRs of the same family (see below). In consequence, the juxtaposition of the cytoplasmic TIR domains recruits the signaling adaptors MyD88 and TIRAP, initiating a signaling pathway that leads to activation of NF-*κ*B transcription factor and MAPK which activate the AP-1 transcription factor [8, 11, 12].

Like other TLRs, TLR2 evolved under strong selection pressure, being preserved in all the vertebrate species tested so far [10, 13]. Sequence information on TLR2 is available for the generality of domestic animals, including cattle, sheep, goat, buffalo, horse, pig, chicken, dog, and cat [14–18].

Species-specific variations in TLR2 have been reported, mainly at the extracellular domains, possibly reflecting adaptation to different microbial environments [19]. Peculiarities in chicken TLRs include the existence of two types of TLR2 (TLR2a and TLR2b) and of TLR1 (TLR1La and TLR1Lb), originated by gene duplication, and the absence of TLR6 [13]. TLR15, apparently unique to avian species, is phylogenetically related to the TLR2 family [13].

### 2.2. Cells and Tissue Expression

TLR2 expression has been reported in antigen presenting cells (APCs), namely, macrophages, monocytes, and dendritic cells (DCs), including CD8*α* ^+^, CD8*α* ^−^, and plasmacytoid DCs in the mouse and interstitial and Langerhans DCs but not plasmacytoid DCs in humans [20]. TLR2 immunomodulatory influence can also be exerted directly on B cells, CD4^+^ and CD8^+^ T cells, T_reg_ cells, *γ* *δ* T cells, natural killer (NK) cells, neutrophils, basophils, and some epithelial cells [21, 22].

Tissue and cell distribution of TLR2 expression in domestic animals follows in general terms what has been described for mice and humans (for comprehensive reviews, see [14, 15]). Most of the information available, summarized in Table 1, has been obtained by reverse transcription (RT)-PCR and data on distribution and levels of the protein itself is sparse due to the lack of characterized specific antibodies for domestic animals. However, in the last few years, efforts have been made to fill this gap and anti-TLR2 antibodies have been used to assess TLR2 expression in different species, namely, bovine and ovine [23], porcine [17, 24, 25], chicken [26], and dogs [27].

In the bovine, differences in TLR2 expression on monocytes, macrophages, and DCs were found by RT-PCR. Monocytes and monocyte-derived macrophages have shown a higher signal, alveolar macrophages and bone marrow-derived DCs an intermediate signal and monocyte-derived DCs, as well as CD172a^+^ and CD172a^−^ DC subsets of afferent lymph, have shown weaker signals [28]. These differences were later confirmed by flow cytometry using anti-TLR2 antibodies [23]. Expression of TLR2 on ovine and bovine peripheral blood mononuclear cells (PBMCs) was detected only in CD14^+^ monocytes [23]. No differences were observed comparing different sheep breeds [23]. Das et al. [29] analysed TLR2 sequences from nilgai, buffalo, sheep, and goat and, interestingly, found that nilgai immune cells and tissues express more TLR2 transcripts than buffalo.

Studying the expression of TLR2 in gut-associated lymphoid tissues from adult swine, Tohno et al. [30] showed that TLR2 mRNA was preferentially expressed in the mesenteric lymph nodes and Peyer's patches in levels higher than that of spleen, and Western blotting confirmed the high TLR2 expression in these structures. Beside immune cells, like T and B cells, TLR2 expression was also detected in membranous cells (M cells). Its detection in the apical membrane of the pocket-like M cells suggests a possible role in ligand-specific transcytosis and transport in these cells.

After raising a panel of monoclonal antibodies for porcine TLR2, Alvarez et al. [24] could demonstrate TLR2 expression in monocytes, macrophages, and granulocytes but not on peripheral blood lymphocytes. TLR2 expression was also detected in nonimmune cells lining body entry sites like tracheobronchial and intestinal epithelial cells, bile ducts in the liver, renal tubules, and basal layer of the epidermis [24]. Expression of TLR2 and TLR6 was demonstrated in porcine alveolar macrophages by Western blot and using antibodies against these receptors, their relevance in the sensing of* Mycoplasma hyopneumoniae* was shown [17]. Also in the horse, TLR2 expression was detected by RT-PCR in alveolar macrophages [31], as well as in respiratory epithelia [32] and PBMCs [33].

Expression of chicken TLR2a and TLR2b was detected in high levels by Western blot in heart, liver, gizzard, and muscle [26] and was also identified by RT-PCR in heterophils, monocytes, macrophages, and B and T cells [34, 35].

Ishii et al. [18] studied the mRNA expression of canine TLR2 in different dog tissues and found it in blood mononuclear cells, lymph node, lung, liver, spleen, bladder, pancreas, small intestine, large intestine, and skin. Bazzocchi et al. [27] found that TLR2 mRNA is constitutively expressed in canine blood neutrophils and, by flow cytometry, it was detected on the blood neutrophils, monocytes, and, at lower levels, lymphocytes.

For the cat, TLR2 expression was reported in lymphoid tissues (spleen and thymus), in lymphocytes (CD4^+^ and CD8^+^ T cells and, in higher levels, CD21^+^ B cells) [36], in bone marrow-derived DCs [37], and in the oral mucosa [38].

### 2.3. The Natural TLR2 Ligands

TLR2 is usually described as the TLR recognizing the largest range of ligands. These include components from bacterial cell walls such as lipoproteins, peptidoglycan (PGN), lipoteichoic acid (LTA), lipopolysaccharides (LPS) from some bacterial species (e.g.,* Porphyromonas gingivalis*), porins from* Neisseria*, lipoarabinomannan from mycobacteria, and zymosan and phospholipomannan from yeast cell walls, among others [6, 8, 39]. The recognition of such a variety of ligands is attributed to the formation of heterodimeric structures with other membrane molecules, like TLR1, TLR6, CD36, CD180/RP105, or dectin-1 [8]. This is however a controversial issue, since some authors argue that bacterial lipoproteins are the only ligands recognized by TLR2 at physiological concentrations [40]. In addition, TLR2 stimulation by most of other ligands was attributed to contamination with lipoproteins [40–42].

Lipoproteins are membrane structural components of bacteria with diverse molecular structure but with a common lipidic modification at an N-terminal cysteine [43, 44]. In the diacylated specimens, that is, lipidated with two fatty acid residues, the modification consists in a di-O-acylated-S-(2,3-dihydroxypropyl) cysteine. The triacylated specimens have a third fatty acid, bound through an amide link to the same N-terminal cysteine. Examples of diacylated forms are the M161Ag lipoprotein of* Mycoplasma fermentans* from which the macrophage-activating lipopeptide (MALP)-2 is derived [45, 46], the LP44 lipoprotein from* Mycoplasma salivarium* from which the fibroblast-stimulating lipopeptide (FSL)-1 is derived [47], and the synthetic lipopeptide di-palmitoyl-S-glyceryl cysteine (Pam_2_C) SK_4_. The Braun's lipoprotein from* Escherichia coli* is the prototype of the outer membrane triacylated lipoproteins from Gram-negative bacteria and some synthetic lipopeptides used as TLR2 stimulators, for example, tri-palmitoyl-S-glyceryl cysteine (Pam_3_C) SK_4_, have a lipid modification analogue to this lipoprotein [48–50]. Other examples of triacylated lipoproteins are OspA from* Borrelia burgdorferi* [51] and the 19 kDa lipoprotein from* Mycobacterium tuberculosis* [52, 53]. The capacity to stimulate through TLR2 of both diacylated and triacylated lipoproteins is conferred by the lipidic N-terminal moiety [54, 55]. The initial studies pointed that diacylated lipoproteins are signalized through TLR2/6 heterodimers, while the triacylated molecules do so through TLR2/1 heterodimers [52, 56–58]. However, later studies suggested that lipopeptide activation through TLR2 may occur independently of TLR1 and TLR6 [59].

In 2007, Jin and collaborators determined by crystallography the structure of the complex TLR1-TLR2-lipopeptide, allowing a structural comprehension of the heterodimerisation induced by the ligand [60]. The binding of the triacylated lipopeptide (Pam_3_CSK_4_) induces the formation of an “m” shaped heterodimer of the TLR1 and TLR2 ectodomains. This dimerisation occurs by the insertion of the two ester-bound fatty acids in a pocket in TLR2 and the insertion of the amide-linked fatty acid in a hydrophobic channel in TLR1 [60]. The role played by the three lipid chains thus explains the incapacity of the diacylated peptide Pam_2_CSK_4_ to dimerise TLR2 and TLR1. On the other hand, Kang et al. [61] showed that TLR2/6 heterodimer has a reduced affinity for triacylated lipopeptides because TLR6 lacks a proper binding site for the amide-bound lipid chain. They also showed that, in the TLR2-TLR6-diacylated lipopeptide complex, the increased hydrophobic area found in the interface of the two dimerised receptors appears to compensate the absence of interaction between a lipid chain and TLR6. For TLR2 ligands without patent hydrophobic regions for TLR2 binding, such as PGN and zymosan, a structural support for the receptor dimerisation is lacking [61]. TLR10 is nonfunctional in the mouse, but in the human, it was shown to form dimers with TLR2, recognizing triacylated lipopeptides and other microbial components [62]. However, this receptor fails to activate typical TLR-induced signaling and its role remains elusive.

In general terms, TLR2 ligands are the same across different vertebrate species; however, some species specificities have been reported. For example, by cotransfection of bovine TLR1 and TLR2 in HEK293 cells, Farhat et al. [63] showed that the ester-bound acid chains of triacylated lipopeptides need to have at least 12 carbon atoms to activate the bovine heterodimer, contrasting with the murine heterodimer that could already be activated by lipopeptides with fatty acids of only 6 carbon atoms. Willcocks et al. [64] showed that, for some TLR2 ligands, bovine primary macrophages or cells transfected with bovine TLRs respond at lower levels than humans.

Irvine et al. [65] cloned equine TLR2, TLR1, and TLR6 and addressed their responses to classical TLR2 ligands. Functionality of TLR2/1 and TLR2/6 heterodimers was demonstrated, with LTA inducing responses similar to those observed with human heterodimers. Pam_2_CSK_4_ activation of TLR2/6 was identical for receptors of both species, while, in opposition to what is observed in human, Pam_3_CSK_4_ was less potent than Pam_2_CSK_4_ in activating the equine TLR2/1 heterodimer.

Different studies addressed the ligand recognition by both types of chicken TLR2 and TLR1 [26, 66, 67]. Together, these studies show that the avian TLR2a and TLR2b form heterodimers with the avian TLR1La and TLR1Lb, allowing the recognition of the same range of ligands that bind the mammalian TLR2 heterodimers, including Pam_3_CSK_4_, MALP-2, FSL-1, and PGN.

## 3. Immunogenic Formulations Targeting TLR2

As occurred with other TLR ligands, the use of lipoproteins and lipopeptides of bacterial origin as adjuvant molecules is well prior to the knowledge of their receptors and mode of action. Soon after the pioneer studies describing and characterizing a lipoprotein present in the cell wall of* E. coli*, published by the groups of Braun and Inouye around 1970 [50, 68–75], it was demonstrated that this lipoprotein, then called Braun's lipoprotein, presented mitogenic properties in mouse B cells [76]. The study of the fragments obtained by hydrolysis of the native lipoprotein enabled Bessler et al. [77] to attribute its mitogenic capacity to the N-terminal triacylated moiety and this was confirmed soon later through the chemical synthesis of lipopeptides with structure analog to the lipid moiety of that region [49]. Following these works, the adjuvant properties of the lipid moiety were tested* in vivo*. The mice inoculation with synthetic lipopeptides covalently bound to a nonimmunogenic peptide of the epidermal growth factor receptor led to the induction of specific antibodies two weeks later after one single administration [78] and the inoculation of guinea pigs with synthetic peptides from the foot-and-mouth disease virus (FMDV) conjugated with the Pam_3_CSS lipopeptide resulted in the induction of neutralizing antibodies and protection against viral infection [79]. At the end of the 1980s, it was reported that the lipopeptides stimulated* in vitro* not only lymphocytes but also human monocytes and mouse macrophages [80] and Deres et al. [81] demonstrated the possibility of inducing* in vivo* CTL responses, restricted to MHC class I, by the inoculation of mice with synthetic lipopeptides conjugated with epitopes from the influenza virus nucleoprotein. Based on these works, as well as in earlier demonstrations of the immunomodulatory effect of protein lipidation [82–85], many studies using synthetic lipopeptides in different disease and immunization models were carried out.

However, it was only at the end of the 1990s that, shortly after the publication describing the cloning and characterization of the human receptor homolog to the* Drosophila* toll [86], different studies reported that TLR2 is a receptor for bacterial lipoproteins [48, 54, 87, 88]. This definitely contributed to understand the adjuvant properties of lipopeptides and lipoproteins and drove a renewed interest in their use as adjuvants. Meanwhile, many other molecules have been claimed to bind TLR2, including nonlipidated molecules, and, although their interaction with the receptor is not entirely elucidated, some have also been used experimentally as adjuvants (e.g., [89, 90]). Among the strategies proposed to explore TLR2 stimulation for the modulation of immune responses, the different systems allowing covalently link lipid moieties to proteic antigens present the most promising applications in vaccinology.

### 3.1. Recombinant Bacterial Lipoproteins Expressed in Fusion with Heterologous Antigens

The first plasmidic vectors for the expression of proteins in fusion with bacterial lipoproteins in* E. coli* aiming at vaccine development were reported in the 1990s. Some of these works had the single objective of exhibiting antigens at the surface of the host bacteria [91, 92], but others were developed also with the purpose of making use of the adjuvant properties of the lipid moiety for the induction of immune responses against heterologous antigens [93, 94]. These vectors consist in partial or complete sequences derived from bacterial lipoprotein genes followed downstream by coding sequences for the heterologous antigens. Among the lipoproteins used as partners in these chimeric structures or used as sources of the lipidation signals are the colicin E2 lysis lipoprotein from an* E. coli* colicinogenic plasmid [94], OprI lipoprotein from* Pseudomonas aeruginosa* [93], Braun's lipoprotein from* E. coli* [95, 96], 26 kDa lipoprotein (Rv1411) from* Mycobacterium tuberculosis* [97], Wza lipoprotein from* Vibrio anguillarum* [98], Ag473 lipoprotein from* Neisseria meningitidis* [99], and OMP19 lipoprotein from* Brucella abortus* [100]. Apart from the Rv1411 lipoprotein from* Mycobacterium tuberculosis*, all the mentioned lipoproteins are originally from Gram-negative bacteria where they are found anchored to the outer membrane. These molecules are first expressed in the cytoplasm as prolipoproteins with an N-terminal signal peptide and are then translocated by the Sec translocon across the inner membrane to the periplasmic side where the processing takes place [101]. The initial lipidation step consists in the binding of a diacylglycerol group through a thioether linkage to the cysteine residue located at the N-terminus of the mature sequence, followed by the cleavage of the signal peptide. The third acyl chain is then attached to the amine group of the same cysteine residue through an amide linkage. Mature lipoproteins are finally transported and anchored on the outer membrane by the Lol System [101, 102]. Due to this maturation process, triacylated forms of the recombinant lipoproteins are only present in the outer membrane of the expression hosts and purification strategies were developed to purify these fully mature forms from the outer membrane [103]. In the cases in which lipoproteins are purified from whole bacterial cell lysates, immature forms, including diacylated lipoproteins, are also present in the final formulations [104, 105].

In some of these cloning and expression systems, multiple cloning sites were included downstream of the lipoprotein gene offering a flexible platform for the cloning of heterologous antigens and were even proposed for shotgun cloning viral genomes and screening for T cell antigens [106]. In some cases, C-terminal hexahistidine tails were also added to enable the purification of the fusion proteins by metal affinity chromatography. When the lipid moieties were characterized, palmitic acid was the predominant fatty acid found, although other fatty acids, including unsaturated, were also present [103, 107].

It is worth mentioning here that there are also examples of vaccine formulations using lipoproteins as homologue antigens, extracted from their native hosts or produced in other expression hosts (e.g., [108, 109]). The classical example is OspA from* Borrelia burgdorferi* for vaccination against Lyme disease [110].

### 3.2. Synthetic Lipopeptides

The chemical synthesis of peptides linked to lipid moieties is another widely used strategy to produce self-adjuvant formulations. Epitopes extended by Pam_3_C or Pam_2_C mimic tri- and diacylated bacterial lipid moieties, but many different variations to this structure have also been developed. These include single-chain palmitoyl-peptides and the more complex lipid core peptide (LCP) and multiple antigen lipophilic adjuvant carrier (MALAC) systems. The covalent attachment of TLR2 agonists to intact proteins has also been reported [111]. Their description and the relations between their structural characteristics and activity have been extensively discussed in detailed reviews (see, e.g., [112–114]) and thus we will not focus on that. However, it is important to stress that differences in the lipid moiety structure, such as the length of the fatty acids and chirality of the glyceryl modification, affect the TLR2-activating properties and this may reflect on the immune response elicited.

Another important point to consider is that some of these synthetic ligands have peptidic and lipid structures very different from the typical bacterial TLR2 ligands and, in certain cases, the dependency on TLR2 activation for their immunomodulatory properties remains to be elucidated. However, for monoacetylated lipopeptides and some other lipoamino acid based lipopeptides the activation through TLR2 is documented [115–117].

## 4. Immunomodulation by Formulations Targeting TLR2

A rational use of adjuvants in the development of better subunit vaccines relies on the understanding of how stimuli exerted at vaccination are translated in specific immune mechanisms, including their magnitude, profile, persistence, and localization. The innate activation through PRRs plays a central role in this shaping of the adaptive immunity and here we resume what has been reported, mainly based on mouse and human studies, concerning TLR2 and TLR2-targeting immunogenic formulations (Table 2).

### 4.1. Modulation of APC Migration and Antigen Internalization

TLR activation has been implicated in the several steps that culminate in the development of specific immune responses, including APC migration. Recently, addressing different adjuvants on influenza subunit vaccines, a role for TLR2 activation in leukocyte migration to inflammation foci was suggested [118]. Pam_3_CSK_4_ was shown to be more efficient than CpG and resiquimod, respectively, TLR9 and TLR7/8 ligands, at inducing an early recruitment of CD11b^+^ blood cells, mainly neutrophils, at the injection site and this observation correlated with the higher capacity of Pam_3_CSK_4_ to enhance antibody responses against influenza antigens [118]. TLR ligands were shown to transiently reduce the motility of DCs at the inflammatory sites, allowing for an extended contact between DCs and the antigen at the site of inflammation [119, 120]. However, the DC activation through TLRs downregulates receptors for inflammatory chemokines and upregulates CCR7 promoting their subsequent migration through lymphatic vessels and localization in the T cell areas of the regional lymph nodes [4, 121–123], and this was reported for TLR2 agonists as well [124, 125].

Internalization of pathogens is also regulated by TLR activation at the inflammatory sites. An initial transitory increasing in the internalization of antigens in response to TLR ligands occurs and is followed by the characteristic reduction in the endocytic capacity of mature DCs, which is congruent with a phenotype specialized in processing and presentation of antigens [126–129].

The above-mentioned transitory reduction in DC motility and the enhancement of antigen internalization was observed upon stimulation of different TLRs including TLR2 [120, 129]. Also demonstrating a TLR2 role in antigen internalization, Schjetne et al. [130] showed that targeting TLR2 with an anti-TLR2 monoclonal antibody leads to the internalization of the ligand into endosomes and its presentation under MHC class II.

### 4.2. Antigen Processing and Presentation in the Context of MHC Class II

Although still controversial [131–133], growing evidences support a determinant role of TLR activation in the control of phagosome maturation, with consequent impact on the regulation of antigenic presentation by APCs. Studies developed by the groups of Blander and of Medzhitov sustain the existence of two modes of phagosome maturation, one constitutive and the other inducible and controlled by TLR signalling [134–136]. According to these studies, the phagocytosis of bacteria leads to a phagolysosomal fusion rate superior to that observed in phagocytosis of apoptotic cells. This difference is only observed in the presence of TLR signalling and involves activation of MAPK p38 through MyD88 activation. Notably, this control of maturation occurs autonomously in the phagosome; that is, the inducible mode is only observed at the phagosomes containing TLR ligands. In addition, TLR activation has been shown to control autonomously the MHC class II loading in the phagosome [136] and to stabilize MHC class II at the cell surface [137]. This autonomous control of phagosome maturation and MHC class II loading may contribute to explain the enhancement of specific immune responses when the antigen is covalently linked to the TLR ligands or is incorporated in the same physical particle [138–141]. The TLR2-targeting formulations in which the antigen is chemically linked to lipid moieties or expressed as fusion with a bacterial lipoprotein ensure TLR activation inside the same phagosomes that contain the antigen and thus are particular interesting tools to modulate antigen processing and presentation. In fact, clearly increased antibody and cellular responses were observed* in vivo* when the antigens were covalently linked to lipopeptides or lipoproteins in comparison to admixed formulations [140, 142, 143].

For the development of an effector response by CD4^+^ T cells, the recognition of a peptide in the context of MHC class II must be accompanied by the engagement of costimulatory molecules expressed at the DC surface, like CD80, CD86, and CD40. In the absence of costimulation, T cells are instructed towards a regulatory or anergic phenotype, resulting in tolerance to the presented antigen [144]. The upregulation of these molecules and of MHC class II at the surface of DCs is generally induced through TLR activation [121]. TLR2 is not an exception. In several studies, the activation of APCs with synthetic lipopeptides and recombinant lipoproteins resulted in cell maturation, with upregulation of MHC and costimulatory molecules (e.g., [99, 142, 145–152]). Moreover, the presentation of MHC class II epitopes to specific T cells by DCs has been demonstrated to be enhanced in the presence of TLR2 agonists [129, 149].

### 4.3. CD4^**+**^ T Cell Polarization

The fate of CD4^+^ T cells is a key issue for the type of immunity elicited upon immunization and its adequacy to the challenge is of capital importance for the success of a vaccine. In this respect, the consequence of TLR activation is not the same for the different TLR ligands and this is probably the most controversial point regarding TLR2 activation and the use of TLR2-targeting formulations in vaccination.

Some authors associate the activation through TLR2 with the induction of Th2 responses [151–155]. According to the model proposed by Dillon et al. [151], the activation via this receptor induces a high level of ERK1/2 signalling resulting in the stabilization of the transcription factor c-Fos suppressing IL-12(p70) production and promoting IL-10 secretion, thus favouring Th2 type responses. Moreover, Gautier et al. [156] attribute the secretion of IL-12(p70) by DCs, essential for Th1 polarization, to an autocrine-paracrine loop of type I interferon (IFN) initiated in response to TLR activation. Stimulation via TLR2 induces the activation of a MyD88-dependent signalling pathway, with activation of NF-*κ*B and the MAPK pathway, resulting, among other effects, in the production of proinflammatory cytokines but not of type I IFN. This would justify the incapacity to polarize responses towards Th1 type via TLR2.

However, other authors pointed TLR2 stimulation or the use of adjuvants composed of TLR2 ligands as an efficient strategy to induce Th1 responses through APC activation [87, 117, 143, 157–163]. Moreover, according to Imanishi et al. [164], the stimulation of mouse Th1 cells by TLR2 ligands directly induces IFN-*γ* production, as well as cellular survival and proliferation in the absence of TCR stimulation. The same is not observed for ligands of other TLR receptors, suggesting an important role of TLR2 activation in the promotion and maintenance of Th1 responses.

In other experimental conditions, the induction of Th17 polarization by IL-1*β*, TGF-*β*, and IL-23 produced by human Langerhans cells stimulated via TLR2 was reported [165]. Synthetic lipopeptides or bacterial lipoproteins have also been shown to induce Th1/T_reg_ differentiation and inhibition of Th2 responses, suggesting a potential application in the treatment of asthmatic diseases [147, 166, 167]. In fact, a regulatory role has been attributed to TLR2 in several studies and its targeting for the induction of tolerogenic responses has also been proposed [168–172, 191]. However, experimental evidences support that the direct activation of T_regs_ by the TLR2/1 ligand Pam_3_CSK_4_, together with stimulation through TCR in the presence of IL-2, induces proliferation and temporary loss of suppressive properties, which is restored after removing the stimulus [173, 174]. Furthermore, the same TLR2 ligand was reported to exert antitumor effects, either through the reduction of the suppressive function of T_regs_ [175] or by the enhancement of the resistance of T effector cells to T_reg_ suppression [176].

The observations supporting divergent TLR2 polarizing properties were obtained from distinct models, using various TLR2 ligands and looking at different levels of the immune response, from the molecular signaling level up to* in vivo* context. As underlined by Mele and Madrenas [192] and supported by other studies [171], it is clear that TLR2 plays both proinflammatory and regulatory roles and that through TLR2 activation, alone or in combination with other stimuli, both effector and regulatory immune mechanisms can be elicited* in vivo* depending on not yet completely clarified factors. To focus on understanding these discrepancies should be a priority for rationally exploring TLR2 stimulation in vaccine development.

### 4.4. Cross-Presentation and CD8^**+**^ T Cell Cytotoxicity

TLR activation has been also implicated in the induction of cross-presentation of antigens by DCs as consequence of enhanced antigen internalization and delivery to the cytosol as well as increase in TAP and proteasome activity [128, 129]. A MyD88-dependent cross-presentation mechanism that requires the dislocation of TAP to the early endosomes was also reported [193], suggesting a spatial separation between endogenous MHC class I-restricted antigen presentation and cross-presentation of exogenous antigens, the latter being biased toward antigens containing PAMPs. Cross-presentation and* in vivo* induction of CTL by TLR activation are usually attributed to TLR ligands high inducers of type I IFN [126, 194, 195], which is not the case of TLR2 ligands. However, different studies demonstrate the induction of these mechanisms by antigens conjugated with TLR2 ligands [140, 146, 157, 177, 178]. In fact, this has been from the beginning one of the most appealing characteristics of these immunogenic formulations [81, 196]. Initially, it was suggested that this capacity could be due to the access of lipidated peptides to the cytoplasm of APC, facilitated by the interaction of the membrane lipids with those of the lipoprotein, with consequent entering in the MHC class I processing pathway [179]. Another explanation resided in the physical properties conferred to the formulations by the lipids, for example, the possible formation of micellar structures and the consequent processing by APCs identical to that observed with particulate antigens [180]. Although these mechanisms are fundamentally of physical nature and TLR-independent, the activation of these receptors also plays an important role in the induction of CTL responses. In fact, Khan et al. [181] showed that *S* and *R* glycerol configurations of Pam_3_CSK_4_ are equally internalized by DCs but diverge at their capacity to induce cytokines and maturation markers on these cells, as well as on the induction of CTL responses* in vivo*. This suggests a determinant role of TLR2 activation on promoting the CTL immune mechanisms. Additionally, it was demonstrated that DC stimulation with the TLR2 ligand MALP-2 induces the expression of proteins of the immunoproteasome LMP2, LMP7, and MECL and an increasing in the proteolytic activity and thus the antigenic processing, suggesting that lipopeptides may indirectly increase the responses restricted to MHC class I [149].

Monoacylated lipopeptides, lipidated through the covalent binding of palmitic acid to the lateral chain of lysine, induce CD8^+^ T cell responses [180, 182] and, although monoacylation do not correspond to the native structure of bacterial lipoproteins, Zhu et al. [117] found that these molecules enhance internalization and DC maturation through TLR2. Zhang et al. [187] also demonstrated the induction of herpes simplex virus (HSV)-2-specific memory CD8^+^ CTL both locally and systemically after intravaginal immunization with a peptide extended by a lipid moiety with three palmitic acids and showed that this response was significantly lower in TLR2^−/−^ and MyD88^−/−^ mice. The inoculation of ovalbumin (Ova) peptides together with BPP, a synthetic derivative of the MALP-2, induced CTL responses which were much higher when the peptides were directly linked to the TLR2 ligand comparing to admixed antigens and adjuvants [150].

### 4.5. Induction of NK Cells Activity

TLR2-dependent NK cell activation was demonstrated to play a role in the immune response against different virus and bacteria [197–201] suggesting the possibility to explore TLR2 ligands as inducers of NK cell activity in therapeutic or prophylactic immunomodulation. The activation of NK cells by different TLR2 agonists has been demonstrated, although the requirement for accessory cells, namely DCs, and accessory cytokines is still debatable [202–204]. Also, variations in the capacity to stimulate NK cells were found among different TLR2 ligands. For example, the activation of NK cells by MALP-2 through stimulation of TLR2 on bone marrow-derived DCs is much less effective than Pam_2_CSK_4_ [184] and the peptide primary sequence in synthetic Pam_2_C lipopeptides has been demonstrated to influence the capacity for NK activation, both directly through TLR2 on NK cells and via DCs [183].

*In vivo*, the subcutaneous injection of Pam_2_CSK_4_ around NK sensitive B16D8 tumor cells led to tumor retardation and this activity was abrogated by injection of asialoGM-1 antibodies, while this antitumor activity was not observed for MALP-2 ligand which was less effective in inducing NK activity [184]. However, in another study, the administration of diacylated lipopeptides (MALP-2 and different Pam_2_ peptides) in mice induced IL-10 and T_reg_ cells that prevented effective antitumor therapeutic responses [172]. The authors also reported the production of IL-10 by NK cells stimulated by the TLR2 agonists* in vitro*. In an antitumor prophylactic immunization, Kiura et al. [154] reported that coadminisration of tumor associated antigens with the diacylated lipopeptide FSL-1 could induce antitumor antibody dependent cellular cytotoxicity (ADCC) by NK cells.

The role of TLR-mediated activation of NK cells in immune responses against infectious and tumor disease is now emerging [205]. TLR2-mediated activation of NK activity for the shaping of the adaptive response, as well as the activation of NK activity in the context of a recall response, will certainly be an interesting research field for the development of future prophylactic vaccines.

### 4.6. Induction of Antibody Responses

Antibody titres, affinity, avidity, and neutralizing capacity are for many diseases the best correlative of protection and to elicit a proper and long-lasting antibody response is thus frequently a desirable achievement in vaccination.

The role of TLR activation in antibody responses and its longevity is a theme of actual research. Pasare and Medzhitov [206] showed that the generation of T-dependent antigen-specific antibody responses requires activation of TLRs in B cells. Kasturi et al. [207] have shown that administration of Ova in synthetic nanoparticles together with the TLR4 ligand monophosphoryl lipid A (MPL) and, simultaneously, with the TLR7 ligand R837 leads to synergistic increases in the anti-Ova antibody response and provides a sustained memory for 1.5 years. This was dependent on DCs, Th cells, and the direct TLR stimulation on B cells.

The specific role of TLR2 in the development of antibody responses is now emerging. TLR2 has been recently demonstrated to be involved in the generation and longevity of antibody secreting cells (ASC) [185] and the addition of CD40 signalling to TLR1/2 and TLR2/6 agonists have been shown to stimulate differentiation of B cells into ASC [186]. Moreover, lipidated formulations targeting TLR2 are widely described as good inducers of antibody responses (e.g., [99, 117, 143, 146, 154, 155, 159]).

### 4.7. Mucosal Immune Responses

Many of the most relevant diseases of humans and veterinary animals are caused by infectious and parasitic agents entering the target hosts through mucosae. Immunization via different mucosal surfaces using TLR2-targeting formulations has been demonstrated to induce strong immune responses, including mucosal IgA and serum IgG as well as local and systemic CD8^+^ CTL [124, 146, 187–189]. Efficient induction of mucosal immune responses is not usually achieved by parenteral administration of vaccines, requiring the presentation of antigens directly at the mucosal surfaces. However, TLR2/1 signals, but not signals from other TLRs, have been shown to be capable of educating extraintestinal DCs with gut-specific imprinting properties [190]. Considering that an immune response elicited apart from the strongly regulatory mucosal environment may be more freely modulated, this capacity of localizing immune responses at the mucosal level through nonmucosal immunization may open the possibility to better tailor the adaptive mucosal immunity. For this purpose, TLR2-targeting formulations are particularly interesting tools.

## 5. Immunomodulation through TLR2 for Veterinary Vaccines

For the veterinary species, the modulatory effect of TLR2 activation on immune response is much less characterized than in humans or in the mouse model. However,* in vitro* activation of APCs or PBMCs by TLR2 ligands was demonstrated for different species as it was the adjuvanticity of TLR2-targeting formulations when inoculated* in vivo*. Evidences on the capacity to elicit CTL responses were also obtained but information on the immune response profiles is sparse. Here, we compile some of the most relevant studies available regarding immunomodulation through TLR2 in veterinary species.

### 5.1. Ruminants

In ovine, stimulation of bone marrow-derived DCs by LTA resulted in upregulation of MHC class II in the CD11b^dull^ subset, which acquired strong capacity of stimulating CD4^+^ T cells in allogenic assays [208]. In bovine, Pam_3_CSK_4_ stimulation of monocyte-derived DC also induced DC maturation, with upregulation of MHC class I, MHC class II, CD40, CD80, CD86, and CD1b molecules, and lead to the production of IL-12 and TNF-*α*. In addition, stimulated DCs promoted IFN-*γ* secretion when cocultured with allogeneic PBMCs [209]. In contrast, the stimulation of macrophages lead to the downregulation of MHC expression and to an almost null effect on IL-12 and TNF-*α* production and on IFN-*γ* secretion in mixed leukocyte reaction [209]. However, Franchini et al. [210] reported that bovine macrophages produce nitric oxide (NO) and TNF-*α* in response to TLR2 activation and that this production is strongly increased by costimulation with IFN-*γ*.

Nelson et al. [211] have shown the possibility to induce active vitamin D_3_ (1*α*,25-dihydroxyvitamin D3) in bovine monocytes through TLR4 and TLR2 activation. Notably, in the mouse, the induction of vitamin D3 in APCs was shown to be related with the imprinting of skin-tropism by APCs in T cells [212]. How this could be exploited for the induction of skin-tropic responses in farm animals remains to be studied. More recently, it was reported that bovine*γ* *δ* T cells directly respond to TLR2 ligands with increased proliferation and cytokine production in a TCR-independent manner [213].

Investigating the potential of* Mycobacterium bovis* antigens to stimulate delayed-type hypersensitivity (DTH) response in cattle, Whelan et al. [214] have shown that the combination of Pam_3_CSK_4_ lipopeptide with the ESAT-6 antigen allowed the induction of DTH in experimentally infected calves which did not occur when the antigen was used alone.* In vitro*, the authors showed the induction of TNF-*α* production by bovine DCs stimulated by Pam_3_CSK_4_ and pointed this induction of proinflammatory cytokines as a possible explanation for what was observed* in vivo*.

Aiming at optimizing the protective efficacy of* Mycobacterium bovis* BCG, Wedlock et al. [215] tested combinations of BCG with culture filtrate proteins formulated with a depot adjuvant and mixed with different stimulatory molecules: MPL, a synthetic mycobacterial phosphatidylinositol mannoside-2 (PIM2) and Pam_3_CSK_4_. Evaluating different pathological and microbiological disease parameters, such as the proportion of animals with tuberculous lesions in the lungs and lymph nodes and the number of* M. bovis* culture-positive lymph nodes, the inclusion of Pam_3_CSK_4_ in the vaccine formulation was shown to induce the best protection.

The potential to use lipopeptides for vaccination against foot-and-mouth disease was tested using seven peptides containing FMDV-specific B-cell linear epitopes from structural and nonstructural proteins, synthesized with a Pam_3_C moiety, and delivered intramuscularly emulsified with Montanide ISA 9 [216]. Twenty days after a single immunization, the animals were challenged and four of the seven immunized animals were protected. No correlation was found between protection and antibody titre or virus-specific proliferation but all protected animals showed a strong T-cell response against at least one of the peptides used for immunization.

Also in cattle, lipopeptides with a palmitic acid coupled to the NH_2_-terminal amino acid and delivered in Freund's adjuvant were used to boost an anti-*Neospora caninum* SRS2 (NcSRS2) DNA immunization [217]. Lipopeptide boosting induced strong immune response, characterized by increased NcSRS2-specific lymphocyte proliferation, IFN-*γ*-secreting cells, and levels of specific IgG1 and IgG2a antibodies. Regarding these parameters, this immunization strategy reproduced the immune response observed against* N. caninum* infection in cattle.

### 5.2. Horse

Stimulation of equine monocytes with Pam_3_CSK_4_ induced the production of TNF-*α*, IL-6, IL-1*β*, and IL-10 [218], and in a whole blood assay TNF-*α*, IL-6, and IL-1*β* were also induced by PGN and LTA [219]. Using equine infectious anemia virus (EIAV) CTL epitopes synthesized on multiple antigenic peptide (MAP) system linked to Pam_3_C, Ridgely and McGuire [220] demonstrated the capacity to stimulate CTL activity* in vitro* on PBMCs obtained from horses of different ELA-A haplotypes chronically infected. The stimulated cells were able to specifically lyse EIAV-infected target cells. In addition, immunization of horses with a Pam_3_C-MAP-CTL epitope induced transitory peptide and virus-specific CTL and, although it neither prevented infection nor affected viral load, it induced a protective effect against development of clinical disease following virus challenge, in which vaccinated horses showed less severe fever and thrombocytopenia and did not develop anemia during the first 2 months after infection [221]. In another approach for anti-EIAV immunization with lipopeptides, Fraser et al. [222] inoculated horses with a pool of peptides containing Th and CTL epitopes extended by a palmitic acid molecule to each of the free NH_2_ groups. The immunized horses showed significant postimmunization proliferative responses to Th peptides but no evident CTL response. After challenge, the immunized group also had a significant increase in the proliferative response to the Th peptides and PBMCs from four of five immunized horses showed CTL activity when stimulated 2 weeks later. Nonetheless, no significant protection was observed considering level and course of viral load, the platelet counts, or fever.

### 5.3. Swine

Immunizing pigs against mouse IgG, an increase in the anti-mouse IgG titres was observed by targeting the antibody to TLR2 [223]. In this work, it was also shown that the* in vitro* proliferative response of PBMCs obtained from pigs immunized with mouse IgG was enhanced when restimulation was performed using an anti-TLR2 mouse monoclonal antibody comparing with restimulation with an isotype-matched control.

Using outer membrane preparations from bacteria expressing African swine fever virus (ASFV) antigens in fusion with the OprI lipoprotein, the entering of the antigens in the class I pathway of antigen presentation and the possibility to identify ASFV epitopes specifically recognised by porcine CTL [196] as well as to stimulate specific CTL activity* in vitro* [106] were demonstrated. In a different study [224], with the purpose to test OprI as an adjuvant for a subunit vaccine against classical swine fever (CSF), it was shown that this lipoprotein activated porcine monocyte-derived DCs, upregulating CD80/86 and MHC class II expression, as well as proinflammatory cytokines. The antigenic restimulation of lymphocytes obtained from CSFV-immune pigs cocultured with autologous monocyte-derived dendritic cells was also enhanced by OprI, as measured by proliferation and IFN-*γ* production.* In vivo*, a subunit vaccine adjuvanted with OprI induced partial protection against CSFV infection but less effective than a water-oil-water adjuvanted vaccine tested in parallel.

### 5.4. Chicken

Stimulation of chicken splenocytes with Pam_3_CSK_4_ upregulated not only Th1-associated cytokines IFN-*γ* and IL-12 but also the Th2-associated cytokine IL-4 [225]. The direct stimulation of chicken CD4^+^ T cells by Pam_3_CSK_4_ also significantly upregulated IFN-*γ* but not IL-4, IL-13, and IL-10 [226]. In a study comparing the effect of three different TLR2 ligands on chicken splenocytes, the results observed suggest different kinetics in the production of proinflammatory cytokines. Pam_3_CSK_4_ induced high IL-1*β* response, while FSL-1 induced an early and prolonged expression of IL-8. The three TLR2 ligands, Pam_3_CSK_4_, FSL-1, and lipomannan, induced a mixed Th profile with upregulation of IFN-*γ*, IL-12, IL-4, and IL-13 [227]. Stimulating chicken monocytes, He et al. [35] demonstrated the induction of iNOS mRNA and of NO production by LTA but not by Pam_3_CSK_4_. Erhard et al. [228] tested the adjuvant effect of Pam_3_CSK_4_ and Pam_3_CS linked to Th epitopes when administered together with different antigens. Although varying with the antigen, the antibody responses were enhanced and in certain cases the combination of more than one adjuvant induced even better responses. Testing two lipoproteins from* Pasteurella multocida* as vaccine antigens, Wu et al. [109] report high protection rates in chicken immunized with* E. coli* expressed lipoprotein E after inoculation in double emulsion adjuvant. OprI lipoprotein was shown to bind* in vitro* and* in vivo* to epithelial cells of the trachea and the small intestine of chickens suggesting its potential use as a carrier for antigen delivery at mucosal surfaces [229].

Based on previous demonstration of the immunostimulatory properties of protozoan HSP70 through TLR2 and TLR4, Zhang et al. [90] investigated if* Eimeria tenella* HSP70 could enhance the immunity elicited by* E. tenella* antigen microneme protein 2 (EtMIC2) against avian coccidiosis. EtHSP70 induced the production of IL-12 and IFN-*γ* in chicken embryo fibroblasts and when inoculated* in vivo* together with EtMIC2 resulted in increased body weight gains, decreased oocyst shedding, and increased antibody responses. Levels of IL-12, IFN-*γ*, and IL-17 were also higher compared with the inoculation of the antigen alone. Chickens immunized with EtHSP70 alone also revealed a protective effect against* E. tenella* infection.

## 6. Concluding Remarks

Vaccination in veterinary animals is a cost-effective strategy to promote animal health and may have an important impact on public health by contributing in reducing the use of antibiotics and controlling zoonotic diseases. The development of new vaccines largely relies on the understanding of how activation of innate immunity through PRRs shapes the subsequent adaptive immune response. The possibility of enhancing antigen presentation by covalently linking TLR2 ligands to the antigen and the particular TLR2 properties at influencing the type and localisation of specific immunity are interesting features that can help at solving some of the present vaccine challenges. However, considering the inconsistencies in results regarding the profile of immune responses, it is of major relevance to address how the specific immune mechanisms elicited upon immunization targeting TLR2 are affected by different factors, such as type of ligand, route of administration, doses, and synergies with other innate stimuli. To extend these studies to the field of veterinary vaccinology further implies to address species-specificities. Clarifying these aspects will allow us in the future to make the innate stimulus adequate for a particular challenge in a given species.

## Figures and Tables

**(a):** 

Human and mouse	Reference
Antigen presenting cells: macrophages, monocytes, and DCs (CD8*α* ^+^, CD8*α* ^−^, and plasmacytoid DCs in mouse; interstitial and Langerhans DCs but not plasmacytoid DCs in humans)	[20]

Lymphocytes: B cells, CD4^+^, and CD8^+^ T cells, Treg cells, *γ* *δ* T cells, and NK cells	
Granulocytes: neutrophils, basophils	[21, 22]
Some epithelial cells	

**(b):** 

Species	Cells and tissues reported to express TLR2	Method^a^	Information on the level of expression	Reference
Bovine	Monocytes	RT-PCR/FC	Strong	[23, 28]
	Monocyte derived-macrophages	RT-PCR/FC		
	Alveolar macrophages	RT-PCR/FC	Intermediate	
	Monocyte derived-DCs	RT-PCR/FC	Weak	
	CD172^+^ DCs	RT-PCR/FC		
	CD172^−^ DCs	RT-PCR/FC		
	CD21^+^ B cells	RT-PCR	No signal	[28]

Ovine	CD14^+^ monocytes from PBMCs	FC		[23]

Nilgai and Buffalo	PBMCs, monocytes, DCs, testes, skin	RT-PCR	Higher in Nilgai than Buffalo	[29]
Buffalo	Kidney, endometrium, bone marrow, trachea	RT-PCR	Higher in endometrium and bone marrow	

Swine	Mesenteric lymph nodes and Peyer's patches	RT-PCR, IHC, FC	Higher than in spleen by RT-PCR	[30]
	Heart, thymus, lung, kidney, skeletal muscle, small intestine	RT-PCR	Lower than in spleen	
	M cells	IHC, FC		
	T and B cells	FC	Higher in T cells than in B cells	
	Monocytes, macrophage, and granulocytes, but not on peripheral blood lymphocytes	FC		[24]
	Epithelial cells lining body entries (Lung, jejunum, kidney, liver)	IHC		
	Alveolar Macrophages	WB		[17]

Equine	PBMCs	RT-PCR		[33]
	Alveolar macrophages	RT-PCR		[31]
	Respiratory epithelial tissues	RT-PCR		[32]

Chicken	Heart, liver, gizzard, muscle	RT-PCR, WB	Strong	[26]
	Spleen, caecal tonsil, bursa, liver	RT-PCR	Strong	[34]
	Heterophils, monocytes, macrophages, B and T cells	RT-PCR		[34, 35]

Canine	Blood mononuclear cells, lymph node, lung, liver, spleen, bladder, pancreas, small intestine, large intestine, and skin	RT-PCR		[18]
	Blood neutrophils	RT-PCR		[27]
	Blood neutrophils, monocytes	FC	Higher levels	
	Lymphocytes	FC	Lower levels	

Feline	Spleen, thymus	RT-PCR		[36]
	CD4^+^ T cells, CD8^+^ T cells, CD21^+^ B cells	RT-PCR	Higher in B cells than in T cells	
	BM-DCs	RT-PCR		[37]
	Palatoglossal mucosa	RT-PCR		[38]

^a^RT-PCR: reverse transcription-PCR; FC: flow cytometry; IHC: immunohistochemistry; WB: Western blot.

**Table 2:** Immunomodulation by TLR2 ligands in mouse and human models.

TLR2 ligand^a^	Species and cells/observations^b^	Reference
*Modulation of APC migration and antigen internalization *		
Recruitment of leukocytes		
Pam_3_CSK_4_	**Mouse**/Recruitment of CD11b^+^ blood cells at injection site, mainly neutrophils	[118]
Transient reduction of DC motility at inflammatory sites		
Pam_3_CSK	**Mouse BM-DCs, spl. DCs**/Podosome disassembly, increasing in cell spreading, switch from podosomes to focal contacts	[120]
Increase in DC antigen internalization		
Pam_3_CSK_4;_ Pam_3_CSK	**Mouse BM-DCs, spl. DCs**/Transiently increased pinocytosis	[120, 129]
Mouse anti-human TLR2 mAb	**Human Mo-DCs**/Internalization of an anti-TLR2 monoclonal antibody into endosomes	[130]
Pam_3_CSK_4_	**Mouse BM-DCs, D1 cells**/TLR2-independent; clathrin- or caveolin dependent; covalent link-dependent	[140]
Promotion of DC migration to regional lymph nodes		
Pam_3_CSK_4_	**Mouse BM-DCs, BM-Mf**/*↘*inflammatory chemokine receptors, *↗*CCR7	[125]

*Antigen processing and presentation in the context of MHC class II *		
Enhanced presentation on MHC class II		
Pam_3_CSK_4_	**Mouse BM-DCs, spl. DCs**/When antigen is coadministered with TLR2 ligand but not sequentially	[129]
MALP-2	**Mouse BM-DCs**/When DCs are loaded with MHC class II Ova peptide in the presence of MALP-2	[149]
Upregulation of costimulatory molecules and MHC class II		
rLipo-D1E3; OprI BLP; PGN, LTA; MALP-2	**Mouse BM-DCs**	[99, 147–149]
[Th]-K(P_2_CSS)-[B]	**D1 cells**	[142]
Pam_3_CSK_4_; 19-kDa and Tp47 LPs	**Human PBMC-DCs**	[145]
[Th]-K(P_2_CSS)-[Tc]	**D1 cells**	[146]
Pam_3_C	**Human Mo-DCs**	[130]
BPPcysMPEG	**Mouse**/ *In vivo*, in CD8*α* ^+^ and CD8*α* ^−^ splenic DC subsets	[150]
*P. gingivalis* LPS	**Mouse**/ *In vivo*, in CD8*α* ^+^ and CD8*α* ^−^ splenic DC subsets	[152]
Pam_3_CSK_4_	**Mouse**/ *In vivo*, in CD11c^+^CD11b^+^ and CD11c^+^CD11b^−^ splenic DC subsets	[151]

*CD4* ^ +^ * T cell polarization *		
Induction of Th2 responses		
Pam_3_CSK_4_	**Human Mo-DCs; Mouse**/*↗*ERK1/2 signalling, stabilization of c-Fos, *↘*IL-12p70, *↗* of IL-10	[151, 153]
FSL-1	**Mouse**/Higher IgG1, lower IgG2a; *↗*IL-10 but not IL-12p70 by splenocytes; *↗*MAPK and c-Fos in splenocytes	[154]
*P. gingivalis* LPS	**Mouse**/*↗*IL-13, IL-5, IL-10 but not IFN-*γ* by specific CD4^+^ T cells; no IL-12p70 by CD8*α* ^+^ DCs	[152]
Pam_3_CSK_4_	**Mouse**/*↗*IL-13, IL-1*β*, GM-CSF, B7RP-1, but low IL-12, IFN-*α*, IL-18, IL-27 by BM-DCs. *In vivo↗*IgE and IgG1 but not IgG2a; *↗*IL-13 and IL-5 but not IFN-*γ* by specific CD4^+^ T cells after restimulation or i.n. challenge	[155]
PGN, Pam_3_C, and zymosan	**Human Mo-DCs**/No IL-12p70 while inducing IL-12p40. Related with the incapacity to induce type I IFN	[156]
Induction of Th1 responses		
K(Pam)-[Th] versus K(Chol)-[Th]	**Mouse**/Pam-LP induced higher IFN-*γ* and IL-2 and lower IL-4 by HSV1-specific CD4^+^ T cells and higher IgG2a/IgG1 ratio	[117]
OprI-COOHgp63 BLP	**Mouse**/*↗*IgG2a; Leishmaniosis protection correlated with IFN-*γ* production	[143]
[Th]-K(P2CSS)-[Tc]	**Human Mo-DCs**/Production of IL-12p70 ≥ than that induced by LPS	[157]
Pam_2_CSK_4_ and Pam_3_CSK_4_	**Human**/*↗*IFN-*γ* in CB-PBMCs co-cultured with allogeneic DCs	[158]
Pam_3_CSK_4_	**Mouse**/*↗*IgG2a, *↘*IgG1 and no IgE after oral immunization with gliadin	[159]
Pam_3_C, 19-kDa, and Tp47 LPs	**Human PBMCs and Mo**/*↗*IFN-*γ* but not IL-4 in PBMCs; anti-IL-12 antibody *↘*T cell proliferation; *↗*IL-12p40 in Mo	[160]
PGN, Pam_3_C, Pam_3_CSK_4_	**Mouse splenocytes**/*↗*IL-12p70 and IFN-*γ* (but less than LPS)	[161]
rlipo-E7m	**Mouse**/*↗*IL-12 in BM-DCs; Higher IFN-*γ* and lower IL-5 by restimulated splenocytes	[162]
19- and 38-kD BLPs	**Human Mo; THP-1 cells**/*↗*IL-12p40 in THP-1 cells; 19-kD lipoprotein *↗*IL-12p70 in Mo	[87]
19 kDa BLP; Tp47, OspA, and 19 kDa LPs; Pam_3_CSK_4_	**Human Mo-DCs and Mo**/*↗*IL-12p40 and IL-10 in Mo; *↗*IL-12p40 and IL-12p70 in Mo-DCs (IL-12p70 higher for the lipoprotein than for the lipopeptides)	[163]
Pam_3_CSK_4_ and MALP-2	**Mouse Th1 cells**/Direct stimulation *↗*IFN-*γ*, cellular survival and proliferation in the absence of TCR stimulation; not observed for ligands of other TLRs	[164]
Induction of Th17 responses		
PGN, Pam_3_CSK_4_, MALP-2	**Human Mo-LC**/MALP-2 and PGN *↗*IL-6, IL-1*β*, and IL-23 by Mo-LC; *↗*IL-17 by allogeneic CD4^+^ T cells cocultured with CD1^+^ Mo-LC stimulated with PGN or Pam_3_CSK_4_	[165]
Induction of Th1/Treg differentiation and inhibition of Th2 responses		
OprI BLP	**Mouse**/*↘*airway eosinophilia, IL-4 and IL-13 after i.n. coadministration of OprI with Ova allergen	[147]
LP40	**Human PBMCs and T cells; Mouse *in vivo* models.**/*↗*IL-10, IFN-*γ* and IL-12 by human PBMCs, both directly and on antigen restimulation; *↘*allergy in different models (*↘*Th2 cells, IgE, and eosinophilia)	[166]
Pam_3_CSK_4_	**Mouse**/*↗*IL-12p35 and IL-10 in BM-DCs and oral myeloid DCs; treated DCs *↗*IFN-*γ* and IL-10 by naïve CD4^+^ T cells; Sublingual administration with antigen in Ova-sensitized mice *↘*airway hyperresponsiveness and Ova-specific IL-5 and IL-10 in cervical lymph nodes	[167]
Regulatory role		
Zymosan	**Human Mo-DCs and Mouse spl. DCs, *in vitro*; Mouse, *in vivo***/*↗*high IL-10, low IL-6 and IL-12p70 by DCs; *In vivo*: low costimulatory molecules on splenic DCs or proinflammatory cytokines, induction of specific T cells secreting IL-10 but little Th1 or Th2 cytokines, and unresponsiveness to challenge with the antigen plus adjuvant (IFA)	[168]
Zymosan	**Mouse spl. DCs**/*↗*Aldh1 and IL-10; leads to metabolize vitamin A and stimulate Treg cells	[169]
Zymosan	**Mouse**/T cells from treated mice showed reduced ability to induce diabetes in NOD-Scid mice	[170]
FSL-1	**Mouse**/*↗*Treg cells in the draining lymph node and *↗* the growth of tumor. Anti-CD25 antibody abrogated the protumor activity of FSL-1	[171]
Pam_2_ lipopeptides	**Mouse**/*↗*Treg cells in a TLR2- and IL-10- dependent manner	[172]
Antiregulatory role		
Pam_3_CSK_4_	**Mouse**/Direct activation of Tregs, with TCR and IL-2 stimulation, induced proliferation and temporary loss of suppressive properties, which are restored after removing the stimulus	[173, 174]
Pam_3_CSK_4_	**Mouse**/*↘*suppressive function of Treg cells; *↗*tumor-specific CTL activity	[175]
Pam_3_CSK_4_	**Mouse**/CD4^+^ effector T cells became resistant to Treg-mediated suppression	[176]

*Cross-presentation and CD8* ^ +^ * T cell cytotoxicity *		
Pam_3_CSK_4_-[Tc]	**Mouse**/Induction of specific CTL responses higher than peptide admixed; *In vitro* cross-presentation of peptides in fusion, but not admixed, and independently of TLR2	[140]
[Th]-K(P_2_CSS)-[Tc]	**HLA-A2kb transgenic Mouse; Human Mo-DCs**/Induction of specific CTL responses. Lipopeptide-pulsed human DCs activated antigen-specific IFN-*γ* production by autologous CD8^+^ T cells	[157]
[Th]-K(P_2_CSS)-[Tc] versus Pam_2_CSS-[Th]-[Tc]	**Mouse**/Branched lipopeptide more potent in the primary response; both induced CTLs and conferred long-term protection against Ova-expressing tumor cells	[177]
[Th]-K(Pam or Chol)-[Tc]	**Mouse**/Induction of CTL responses by i.n. route and enhanced protection against influenza challenge	[178]
Pam_3_CSS-[Tc]	**Mouse**/Specific CTL activity induced *in vivo* against influenza virus	[81]
Hda-[Tc]	**Mouse**/Specific CTL activity induced *in vivo* against HIV-1 virus	[179]
K(Pam)-[Tc]	**Human Mo-DCs**/Lipopeptides but not the peptide were endocytosed; *↗*IFN-*γ* of matched specific CD8^+^ T-cells	[180]
Pam_3_CSK_4_-[Tc]	**Mouse**/Induced *in vivo* tetramer positive and IFN-*γ* producing CD8^+^ T cells	[181]
MALP-2	**Mouse BM-DCs**/*↗*immunoproteasome proteins LMP2, LMP7, and MECL; *↗*proteolytic activity	[149]
(K(Pam))_1,2,or 3_-[Th]-[Tc]	**Mouse**/*↗*specific antiviral CTL responses irrespective of the number of lipid moieties	[182]
BPPcysMPEG	**Mouse**/*↗*specific CTL response in a TLR2 and CD4^+^ Th dependent manner, but independent of IFN-*α*. Direct link of the antigen to BPP *↗*CTL	[150]
FSL-1	**Mouse**/Immunization of FSL-1 and tumor-associated antigens *↗*specific CTLs	[171]
rlipo-E7m	**Mouse**/*↗*CTL responses and therapeutic and prophylactic protection against tumor challenge	[162]

*Induction of NK cells activity *		
Stimulation of NK cell activity		
Pam_2_C lipopeptides	**Mouse**/Variations in the activation capacity among lipopeptides with different peptide sequences	[183]
Pam_2_CSK_4_ versus MALP-2	**Mouse**/MALP-2 much less effective in inducing NK cell activity	[184]
Induction of ADCC by NK		
FSL-1	**Mouse**/Coadministration with tumor associated antigens *↗*antitumor ADCC by NK cells	[171]

*Induction of antibody responses *		
Induction of antibody secreting cells (ASC) differentiation		
Natterins	**Mouse**/Generation of long-lived ASCs	[185]
Pam_2_CSK_4_; Pam_3_CSK_4_	**Mouse**/Together with CD40 signal, *↗*differentiation of B cells into ASCs	[186]
Increasing of antibody responses		
Fusion lipoproteins	**Mouse**	[99, 143]
Synthetic lipopeptides	**Mouse**	[117, 146, 154, 155]

*Induction of mucosal responses *		
Immunization via mucosal surfaces		
((Pam)K)_3_-[Th]-[Tc]	**Mouse**/Intravaginally delivery *↗*HSV-2-specific memory CTLs locally and in the spleen. Significantly weaker response observed in TLR2^−/−^ mice	[187]
MAP-Pam_3_C	**Mouse**/*↗*specific IgA in mucosal secretions and IgG in the serum after oral immunization. Intragastric delivery *↗*systemic T cell stimulation and specific CTL activity	[188]
LT-IIa-B5	**Mouse**/i.n. immunization recruited DCs to the NALT, *↗*antigen uptake, CCR7 and migration to draining lymph node. TLR2-dependent *↗* of specific CD4^+^ T cell proliferation, salivary IgA, and serum IgG	[124]
Pam_3_CSK_4_	**Mouse**/i.n. immunization enhanced serum antibody responses and protection against influenza	[189]
[Th]-K(P_2_CSS)-[Tc]	**Mouse**/i.n. immunization induced CD8^+^ CTL responses in the lung and systemic after influenza challenge	[146]
Mucosal imprinting of specific immunity		
Pam_3_CSK_4_	**Mouse**/Pretreatment of extraintestinal DCs *↗*retinal dehydrogenases and confer the capacity to induce gut-homing lymphocytes	[190]

^a^rLipo-D1E3 and rlipo-E7m: Domain 1 of the Ag473 lipoprotein from *Neisseria meningitidis* fused to a sequence of dengue virus envelope protein (E3) or inactive E7 oncoprotein of human papillomavirus (E7m); OprI or OprI-COOHgp63 BLPs: outer membrane lipoprotein I from *Pseudomonas aeruginosa*, alone or fused with a truncated 32-kDa version, the major cell surface glycoprotein gp63 of *Leishmania major*; [Th]: CD4^+^ T cell epitope; [Tc]: CD8^+^ T cell epitope; [B]: B cell epitope; K: Lysine with a palmitic (Pam), diacylated (P2CSS), or cholesterol (Chol) moiety attached to *ε*-amino group; 19-kDa, Tp47, and OspA LP: synthetic tripalmitoyl lipopeptides based on the sequences of 19-kDa lipoprotein from *Mycobacterium tuberculosis*, 47-kDa lipoprotein from *Treponema pallidum*, and OspA lipoprotein from *Borrelia burgdorferi*; 19-kDa and 38-kD BLP: 19-kDa and 38-kD lipoproteins from *Mycobacterium tuberculosis*; LP40: synthetic lipopeptide CGP 40774; Hda: *α*-aminohexadecanoic acid; BPPcysMPEG: pegylated synthetic derivative of MALP-2; MAP: multiple antigen peptide; LT-IIa-B5: pentameric B subunit of type IIa enterotoxin of *Escherichia coli*.

^b^ *↗*: enhancement; *↘*: decrease; BM-DCs: bone marrow-derived DCs; spl. DCs: splenic DCs; Mo-DCs: monocyte-derived dendritic cells; Mo: monocytes; Mo-LC: monocyte-derived Langerhan-like cells; D1 cells: a line of immature DCs derived from spleen cells; PBMC-DCs: PBMC-derived DCs; CB-PBMCs: cord blood-derived PBMCs; THP-1 cells: human monocytic cell line; i.n.: intranasal.

## Abbreviations

ADCC: Antibody dependent cellular cytotoxicity
ASC: Antibody secreting cells
APC: Antigen presenting cell
CTL: Cytotoxic T lymphocyte
DC: Dendritic cell
EIAV: Equine infectious anemia virus
FMDV: Foot-and-mouth disease virus
LPS: Lipopolysaccharide
LTA: Lipoteichoic acid
MPL: Monophosphoryl lipid A
NO: Nitric oxide
NK: Natural killer
OprI: Outer membrane lipoprotein I
Ova: Ovalbumin
Pam_2_C: Di-palmitoyl-S-glyceryl cysteine
Pam_3_C: Tri-palmitoyl-S-glyceryl cysteine
PNG: Peptidoglycan
PRR: Pattern recognition receptor
TLR: Toll-like receptor.
